# miR-101-3p-mediated role of PDZK1 in hepatocellular carcinoma progression and the underlying PI3K/Akt signaling mechanism

**DOI:** 10.1186/s13008-023-00106-6

**Published:** 2024-03-26

**Authors:** Huihui Gao, Zhaofeng Gao, Xiaobei Liu, Xu Sun, Zhonghui Hu, Zhengwei Song, Cheng Zhang, Jianguo Fei, Xiaoguang Wang

**Affiliations:** 1 Department of Internal Medicine, The No.1 People’s Hospital of Pinghu City, Pinghu, 314201 Zhejiang China; 2 Department of Surgery, The Second Affiliated Hospital of Jiaxing University, No. 397, Huangcheng North Road, Jiaxing, 314000 Zhejiang China; 3 Faculty of Graduate Studies, Zhejiang Chinese Medical University, Hangzhou, 310053 Zhejiang China; 4 School of Medicine, Huzhou Central Hospital, Affiliated Huzhou Hospital, Zhejiang University, Huzhou, 313003 Zhejiang China; 5 Department of Hepatobiliary and Pancreatic Surgery, The First Affiliated Hospital, Zhejiang University School of Medicine, Hangzhou, 310003 China

**Keywords:** PDZK1, miR-101-3p, Hepatocellular carcinoma, PI3K/AKT, Cell proliferation

## Abstract

**Background:**

The molecular targets and associated mechanisms of hepatocellular carcinoma (HCC) have been widely studied, but the roles of PDZK1 in HCC are unclear. Therefore, the aim of this study is to explore the role and associated mechanisms of PDZK1 in HCC.

**Results:**

It was found that the expression of PDZK1 in HCC tissues was higher than that in paired paracancerous tissues. High expression of PDZK1 was associated with lymph node metastasis, degree of differentiation, and clinical stage. Upregulation of PDZK1 in HCC cells affected their proliferation, migration, invasion, apoptosis, and cell cycle, and also induced PI3K/AKT activation. PDZK1 is a downstream target gene of miR-101-3p. Accordingly, increase in the expression of miR-101-3p reversed the promotive effect of PDZK1 in HCC. Moreover, PDZK1 was found to accelerate cell proliferation and promote the malignant progression of HCC via the PI3K/AKT pathway.

**Conclusion:**

Our study indicated that the miR-101-3p/PDZK1 axis plays a role in HCC progression and could be beneficial as a novel biomarker and new therapeutic target for HCC treatment.

**Supplementary Information:**

The online version contains supplementary material available at 10.1186/s13008-023-00106-6.

## Background

Hepatocellular carcinoma (HCC) is a malignant tumor that originates from hepatocytes. HCC occurs all over the world, and accounts for more than 90% of primary liver tumors [1, 2]. HCC is a malignant tumor with one of the highest incidence and mortality rates [2, 3]. Every year, more than 600,000 patients are diagnosed with HCC all over the world, and 10% of them die of HCC [4]. The main cause of HCC is currently considered to be viral infections (such as hepatitis B virus or C virus infections), non-viral factors (such as alcohol and aflatoxin), hereditary hepatopathy, and cirrhosis [5, 6]. Although curative therapies are available for HCC, such as liver transplantation, surgical resection, radiofrequency ablation, transarterial chemoembolization (TACE), and sorafenib, the overall long-term survival rate remains poor [7, 8]. Therefore, it is crucial to investigate and elucidate the occurrence, development, and potential molecular mechanism of HCC. Although the pathogenesis of HCC has not been fully elucidated, the abnormal inactivation of tumor suppressor genes and over-activation of oncogenes are considered to be the main factors that cause HCC [9].

PDZK1 (PDZ domain containing 1) is a scaffold protein with four PDZ domains that belongs to the sodium hydrogen exchange regulatory factor family [10]. This family is a recognized family of adaptor/scaffold proteins that plays a role in interactions between a variety of diseases and proteins by targeting and recruiting various factors [11–13] and is mainly expressed in the liver, kidney, pancreas, and adrenal gland. PDZK1 is also a cell membrane adaptor protein that regulates interactions between a large number of proteins [14] and plays a role in cell–cell interaction, cell differentiation, growth regulation, and signal transduction [10]. PDZK1 was originally found in the apical brush border membrane of the proximal tubules of the kidney [15], and in recent years, it has also been found to play a role in high-density lipoprotein transport, esophageal cancer, renal cell carcinoma, small intestine, estrogen-related pigmentation, and ER( +) breast cancer [16–20].

At present, microRNAs (miRNAs) are deemed to be vital regulators of mRNA and influence tumor progression through their effects on the differentiation, cell cycle, and apoptosis of cells [21]. Numerous studies have revealed that targeting miRNAs may be an alternative strategy to control or inhibit tumor progression. For example, the miRNA miR-101-3p was found to be involved in diverse cancers as a suppressor [22, 23]. Further, miR-101-3p was found to suppress the progression of non-small cell lung cancer by inhibiting the PI3K/AKT signaling pathway [23], and it also blocked the progression of bladder cancer by regulating the SPRY4-IT1/EZH2 axis [24]. In serous ovarian cancer, miR-101-3p was found to inhibit epithelial–mesenchymal transition and tumor invasion and metastasis by regulating the PTAR/ZBE1 axis [25]. miR-101-3p has also been found to be involved in HCC progression and radiosensitivity [26, 27]. However, there is very little research about the interaction between PDZK1 and miR-101-3p in HCC. Therefore, this study aimed to explore the roles of and interactions between PDZK1 and miR-101-3p in HCC, in order to identify novel targets for HCC treatment.

## Results

### High expression of PDZK1 in HCC tissues and cell lines and association of PDZK1 expression with the clinicopathological features of HCC

**Fig. 1:**
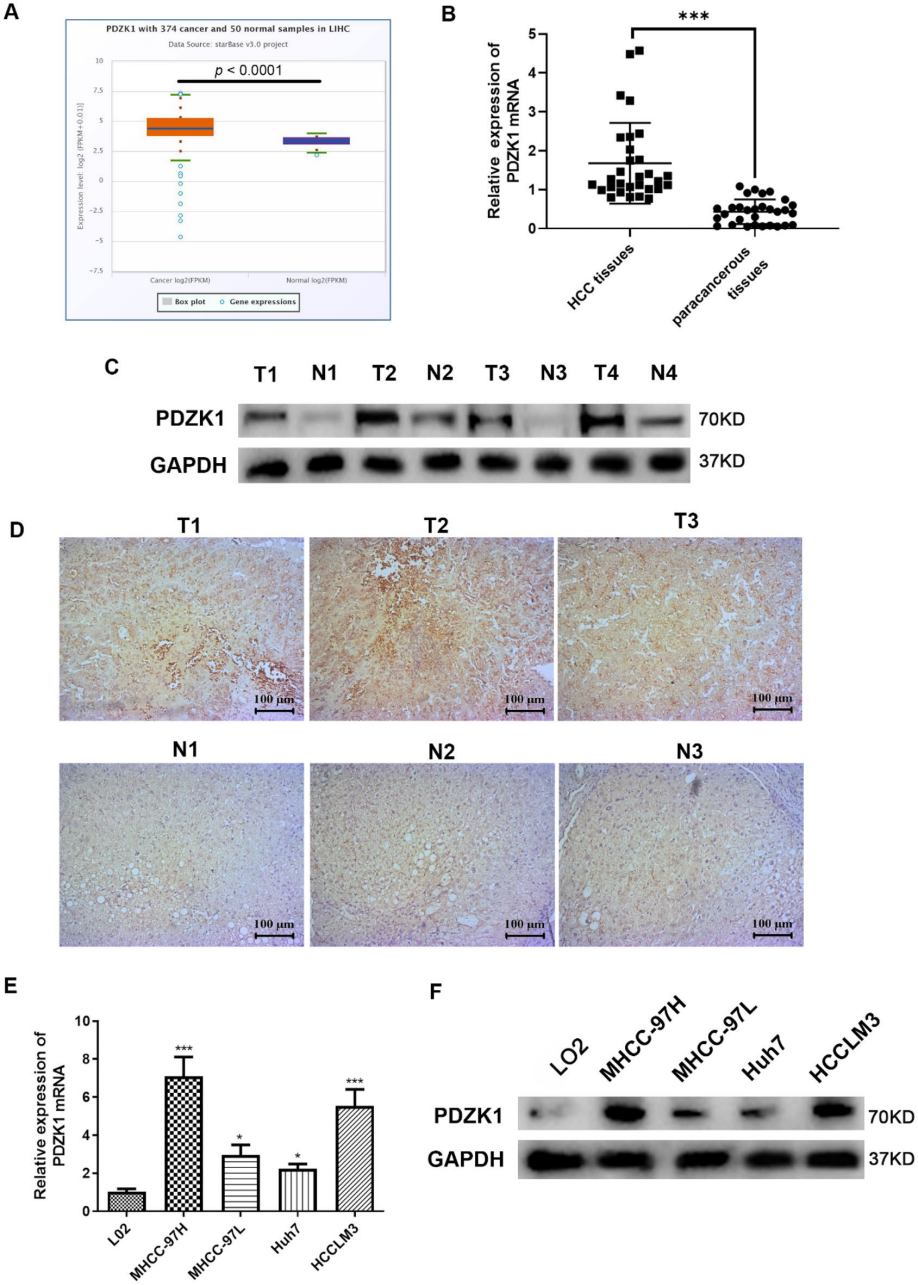
Upregulation of PDZK1 in HCC tissues and cell lines. **A** Data analysis showing higher PDZK1 expression in HCC samples than in normal tissue samples from the TCGA database in Starbase (http://starbase.sysu.edu.cn/). **B** PDZK1 mRNA expression in HCC tissues and paracancerous tissues. **C** Protein expression of PDZK1 in HCC tissues and paracancerous tissues. **D** IHC results for PDZK1 expression in HCC tissues and normal tissues. **E** PDZK1 mRNA expression in HCC cell lines and normal liver cells. **F** Protein expression of PDZK1 in HCC cell lines and normal liver cells. T: Tumor; N: Normal. *P < 0.05, ***P < 0.001

To explore the role of PDZK1 in the development of HCC, data on PDZK1 expression in HCC tissues and normal tissues were obtained from the Starbase database (http://starbase.sysu.edu.cn/). The data demonstrated that PDZK1 expression was significantly upregulated in HCC samples (Fig. 1A). PDZK1 expression was also examined in the HCC tissues and normal tissues collected from the patient cohort. PDZK1 expression was found to be highly upregulated in HCC tissues compared to paracancerous tissues (*P* < 0.01, Fig. 1B, C). The IHC results also indicated obvious upregulation of PDZK1 in HCC tissues compared to normal tissues (Fig. 4D). As shown in Table 1, the PDZK1 level in HCC tissues was closely associated with lymph node metastasis, the degree of tumor differentiation, and clinical stage. Further, PDZK1 expression was higher in the HCC cell lines than in the normal liver cell line (Fig. 1E, F). All these results are in agreement and imply that PDZK1 is a potential tumor promoter gene in HCC. Importantly, high PDZK1 expression may be an important marker for the development of HCC.

**Table 1:** Analysis of the association of PDZK1 expression with the clinicopathological features in 30 HCC patients

Characteristics	No. of cases	PDZK1 expression		*P*-value
		High (≤ medium)	Low (≥ medium)	
Number	30	15	15	
Age (y)				0.7125^a^
< 60	13	7	6	
> 60	17	8	9	
Gender				0.1432^a^
Female	14	9	5	
Male	16	6	10	
TNM stage				< 0.05^a^
I–II	18	6	12	
III–IV	12	9	3	
Degree of differentiation				< 0.01^a^
Well/moderate	17	5	12	
Poor	13	10	3	
Lymph node metastasis				< 0.01^a^
No	19	6	13	
Yes	11	9	2	

^a^ Two-sided chi-squared test

### PDZK1-mediated regulation of the proliferation, migration, and invasion ability of HCC cells

**Fig. 2:**
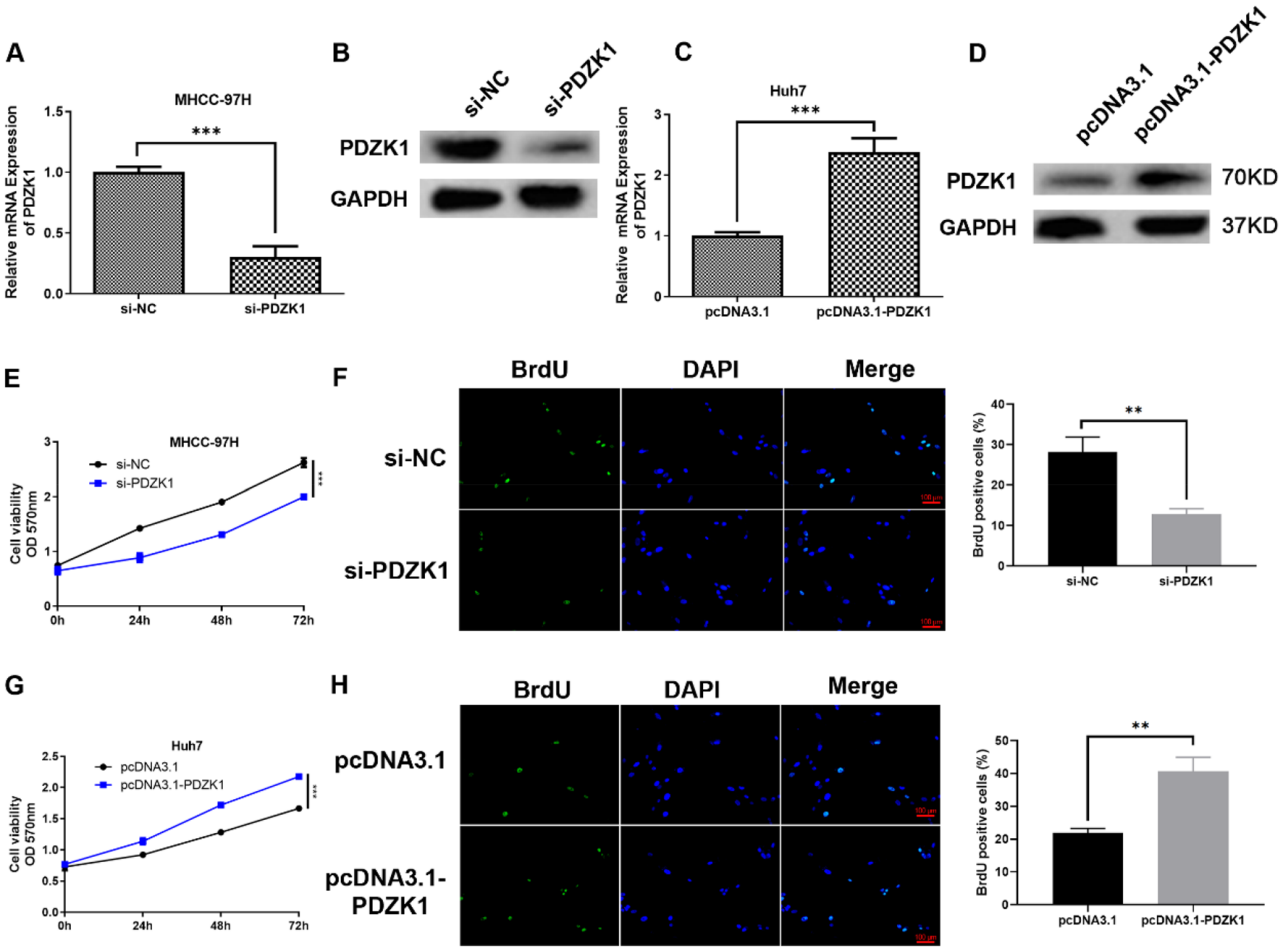
PDZK1-mediated regulation of the proliferation ability of HCC cells. Transfection efficiency of si-NC and si-PDZK1 determined by qRT-PCR (**A**) and western blot analysis (**B**) in MHCC-97H cells. Transfection efficiency of pcDNA3.1 and pcDNA3.1-PDZK1 determined by qRT-PCR (**C**) and western blot analysis (**D**) in Huh7 cells. MTT assay (**E**) and BrdU incorporation assays (**F**) for the evaluation of MHCC-97H cell proliferation. MTT assay (**G**) and BrdU incorporation assays (**H**) for the evaluation of Huh7 cell proliferation. Data are shown as the mean ± SD values (n = 3). **P < 0.01, ***P < 0.001

In order to explore further the influence of PDZK1 on HCC cells, MHCC-97H cells were employed for PDZK1 knockdown experiments and Huh7 cells were used for PDZK1 overexpression experiments, based on the expression of PDZK1 in the HCC cell lines observed in the previous experiments. The PDZK1 level in the si-PDZK1 group, that is, the PDZK1-knockdown group, was significantly decreased (*P* < 0.001, Fig. 2A, B), while the level in the pcDNA3.1-PDZK1 group, that is, the PDZK1-overexpression group, was significantly increased (*P* < 0.001, Fig. 2C, D).

**Fig. 3:**
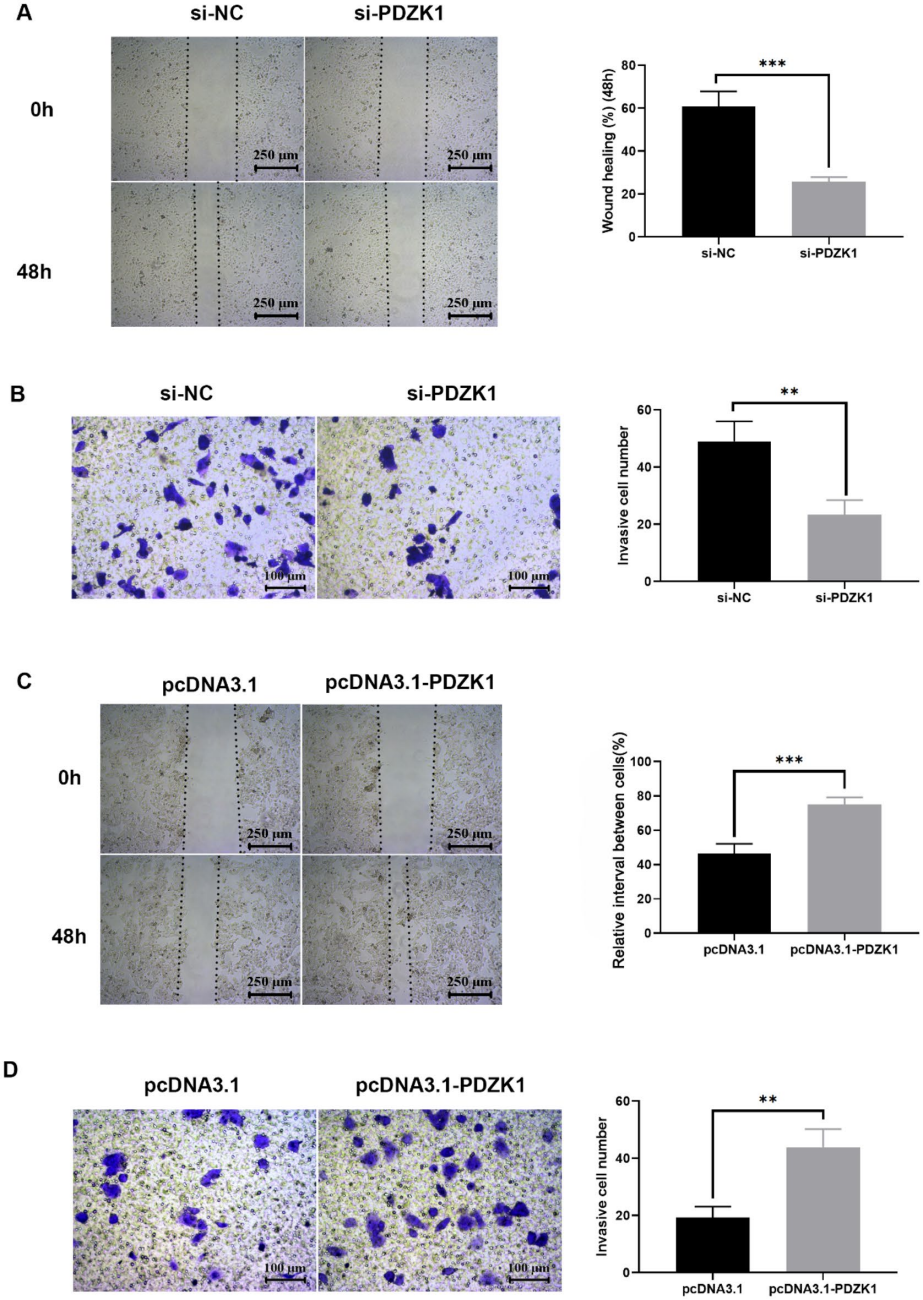
PDZK1-mediated regulation of the migration and invasion ability of HCC cells. **A** Scratch assay of MHCC-97H cell migration. **B** Transwell assay of MHCC-97H cell invasion. **C** Scratch assay of Huh7 cell migration. **D** Transwell assay of Huh7 cell invasion. Data are expressed as the mean ± SD values (n = 3). **P < 0.01, ***P < 0.001

A series of functional experiments were then performed. Proliferation of tumor cells is a major indicator of malignant phenotype. Therefore, the influence of PDZK1 on HCC cell proliferation was investigated using the MTT and BrdU assays. As expected, PDZK1 knockdown significantly inhibited HCC cell proliferation (Fig. 2E, F), while PDZK1 overexpression significantly elevated the proliferation of HCC cells (Fig. 2G, H). Next, the effect of PDZK1 on the migration or invasion of HCC cells was assessed by scratch and Transwell chamber assays. In these experiments, too, PDZK1 knockdown was found to significantly inhibit tumor cell migration (Fig. 3A) and invasion (Fig. 3B) in MHCC-97H cells, while overexpression of PDZK1 promoted the migration and invasion of Huh7 cells (Fig. 3C, D).

### PDZK1-mediated modulation of cell cycle and apoptosis in HCC cells

**Fig. 4:**
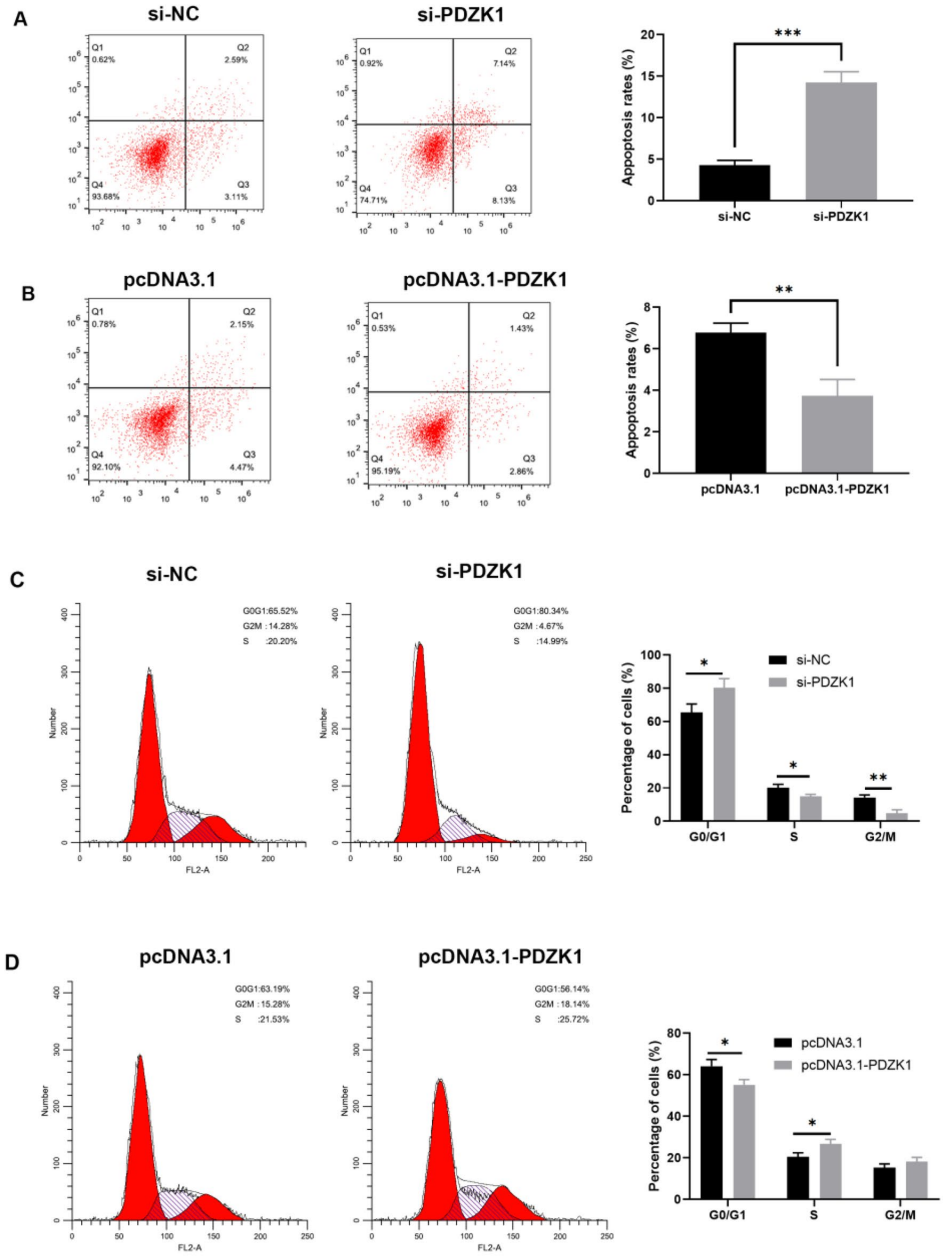
PDZK1-mediated modulation of cell apoptosis and cell cycle arrest in HCC cells. Cell apoptosis assay results for different treatments in MHCC-97H cells (**A**) and Huh7 cells (**B**). Cell cycle assay results for different treatments in MHCC-97H cells (**C**) and Huh7 cells (**D**). Data are expressed as the mean ± SD values (n = 3). *P < 0.05, **P < 0.01, ***P < 0.001

The influence of PDZK1 on apoptosis and cell cycle progression in HCC cells was further investigated with flow cytometry. The apoptosis rate [28] in si-PDZK1-treated MHCC-97H cells was significantly higher than that in si-NC-treated MHCC-97H cells (*P* < 0.001, Fig. 4A). Accordingly, PDZK1 overexpression in Huh7 cells resulted in a significant decline in apoptosis (*P* < 0.01, Fig. 4B). These results demonstrate that PDZK1 knockdown can promote apoptosis of HCC cells.

The cell cycle study demonstrated that downregulation of PDZK1 expression resulted in an increase in the percentage of MHCC-97H cells in the G0/G1 phase (*P* < 0.05, Fig. 4C) and a significant decrease in the distribution of cells in the G2/M and S phase (Fig. 4C). In contrast, overexpression of PDZK1 promoted the transition of Huh7 cells from the G0/G1 phase to the S phase (Fig. 4D). These findings indicate that downregulation of PDZK1 induced cell cycle arrest of HCC cells in the G0/G1 phase.

### Activation of the PI3K/AKT pathway by PDZK1 in HCC cells

**Fig. 5:**
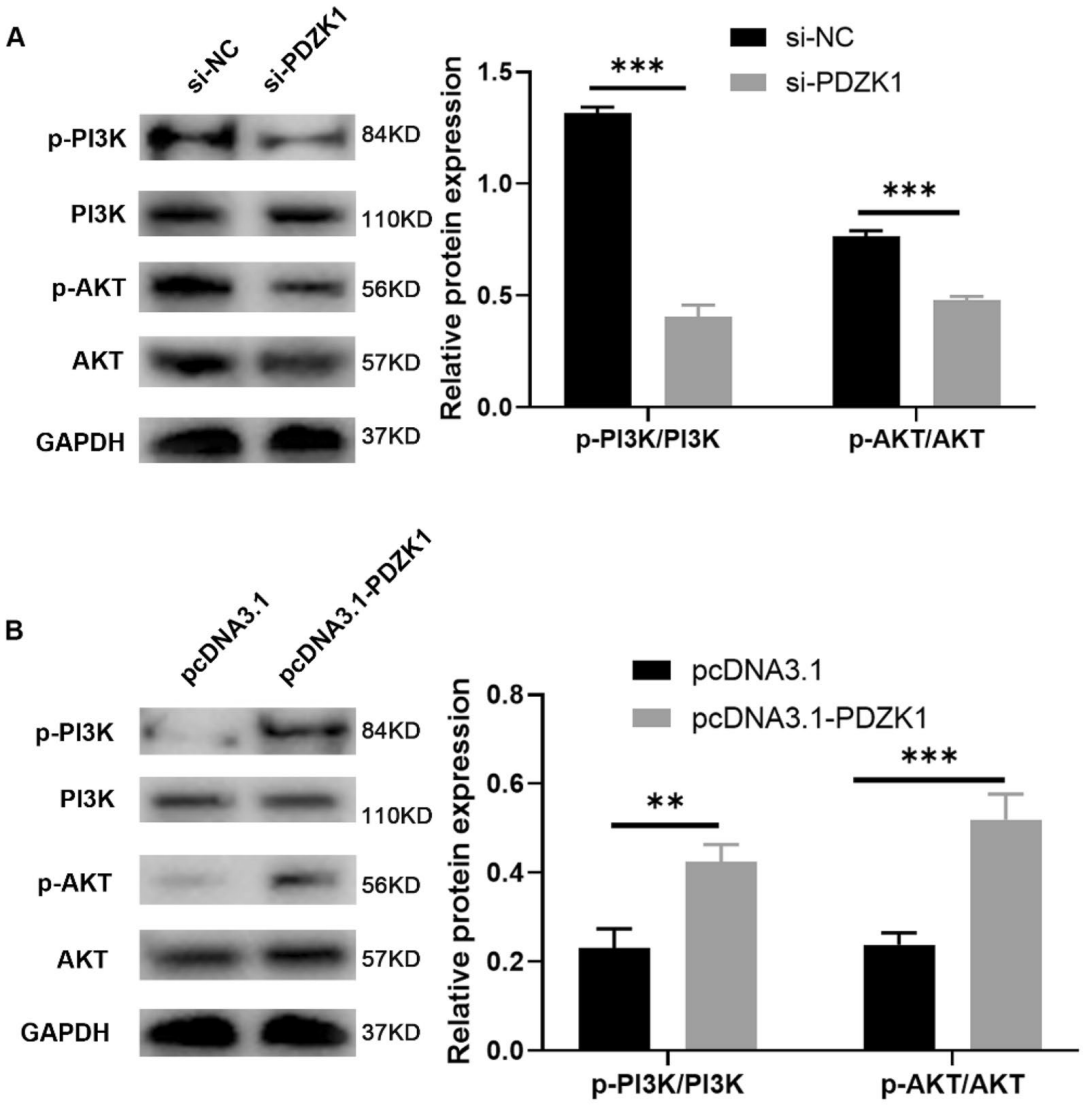
PDZK1-mediated activation of the PI3K/AKT pathway in HCC cells. Protein expression of p-PI3K, PI3K, p-AKT, and AKT in MHCC-97H cells (**A**) and Huh7 cells (**B**) were determined by western blot. Data are expressed as the mean ± SD values (n = 3). *P < 0.05, **P < 0.01, ***P < 0.001

It has been reported that PDZK1 is associated with the PI3K/AKT signaling pathway [29]. Therefore, to explore the possible molecular mechanisms of PDZK1 and its relevant pathways in HCC, we performed western blot analysis of key proteins involved in PI3K/AKT signaling in PDZK1-knockdown and PDZK1-overexpression HCC cells. The results showed that phosphorylation of AKT or PI3K was decreased after PDZK1 knockdown (Fig. 5A), while the level of p-AKT and p-PI3K was increased after PDZK1 overexpression (Fig. 5B). Thus, higher expression of PDZK1 was associated with an increase in the phosphorylation of AKT and PI3K, and this implies that PDZK1 is involved in the activation of the PI3K/AKT pathway.

### PDZK1 as a direct target of miR-101-3p

**Fig. 6:**
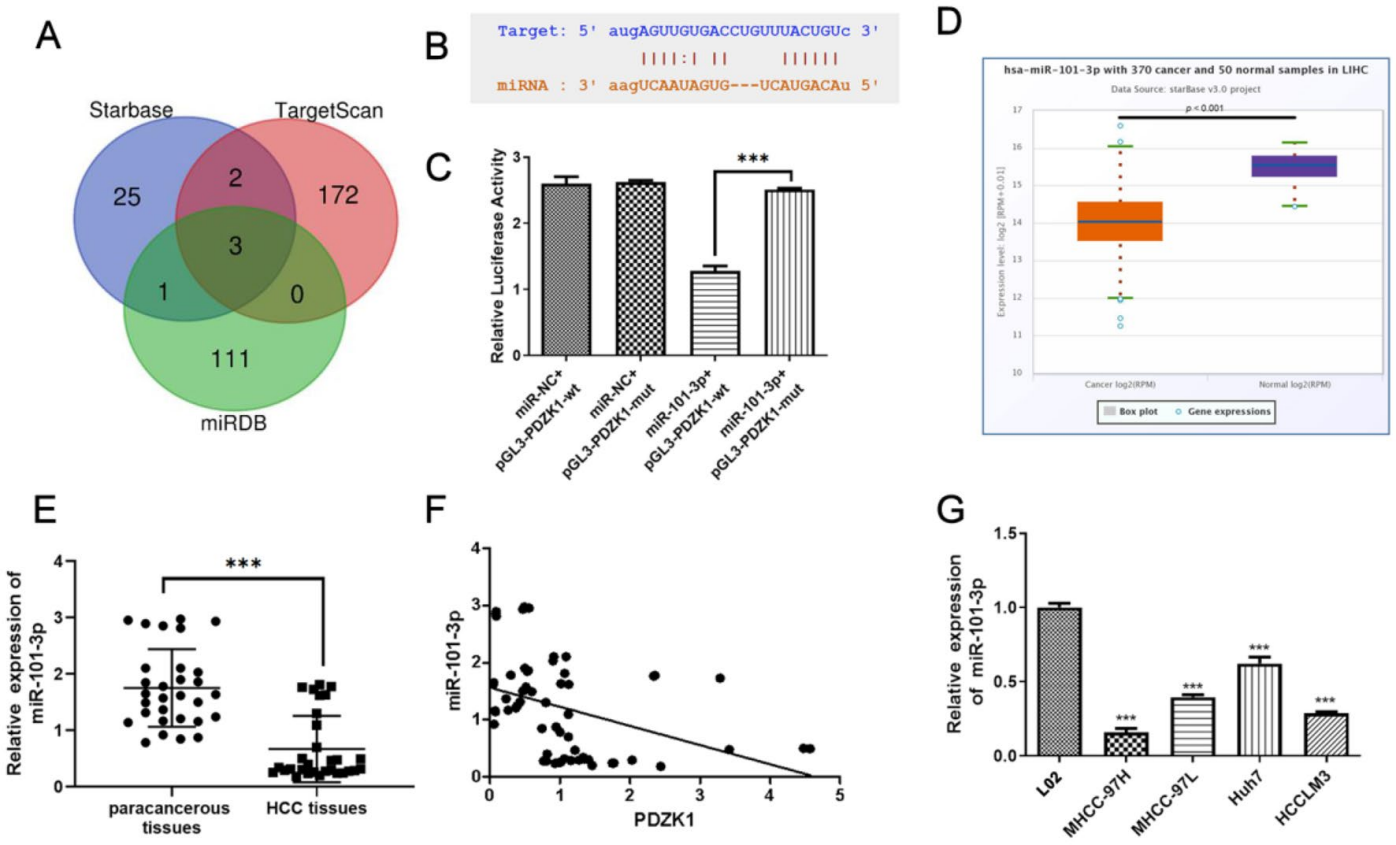
Interaction between miR-101-3p and PDZK1. **A** The Venn diagram represents overlapping miRNAs, as predicted by the miRDB, Starbase, and TargetScan databases. **B** Binding sites of miR-101-3p and PDZK1. **C** Dual-luciferase assay of miR-101-3p and PDZK1. **D** miR-101-3p expression in HCC samples from the TCGA database in Starbase (http://starbase.sysu.edu.cn/). **E** miR-101-3p expression in HCC tissues and paracancerous tissues. **F** The negative relationship between PDZK1 and miR-101-3p expression in HCC tissues and paracancerous tissues can be observed. **G** miR-101-3p expression in HCC cell lines and normal liver cells. Data are expressed as the mean ± SD values (n = 3). ***P < 0.001

The interaction between mRNAs and miRNAs is considered to be a vital factor in the development of tumors. The Starbase, TargetScan, and miRDB databases were utilized for prediction of the target miRNAs of PDZK1 (Fig. 6A). Three candidate miRNAs were considered, namely, hsa-miR-30a-5p, hsa-miR-2114-3p, and hsa-miR-101-3p. Next, we compared the levels of these miRNAs in the Starbase and clinical samples. As shown in Figure S1 (Additional file 1), miR-30a-5p had lower levels of expression in HCC tissues than in normal tissues in the Starbase samples, but the opposite trend was observed in samples obtained from our hospital. However, miR-2114-3p was expressed at higher levels in HCC tissues than in normal tissues in the Starbase samples, while there was no significant difference in the samples from our hospital. As observed for miR-30a-5p, miR-101-3p had significantly lower expression levels in HCC tissues than in normal tissues in the Starbase samples; moreover, this trend was also observed in the samples from our hospital. Based on these findings, we selected miR-101-3p for the following studies. The binding sites between PDZK1 and miR-101-3p are depicted in Fig. 6B, and their interaction effect was demonstrated by the luciferase reporter assay (Fig. 6C). Relative luciferase activity was lower in the miR-101-3p + pGL3-PDZK1-wt group than in the miR-101-3p + pGL3-PDZK1-mut group. Further, the miR-101-3p level was low in HCC samples from the Starbase database (Fig. 6D) and from the samples we collected (Fig. 6E). As shown in Fig. 6F, a negative relationship was observed between miR-101-3p and PDK1 levels in samples from the current cohort. In addition, miR-101-3p expression was significantly lower in HCC cells than in normal liver cells (Fig. 6G).

### miR-101-3p-mediated effect of PDZK1 on the proliferation, migration, and invasion ability of Huh7 cells

**Fig. 7:**
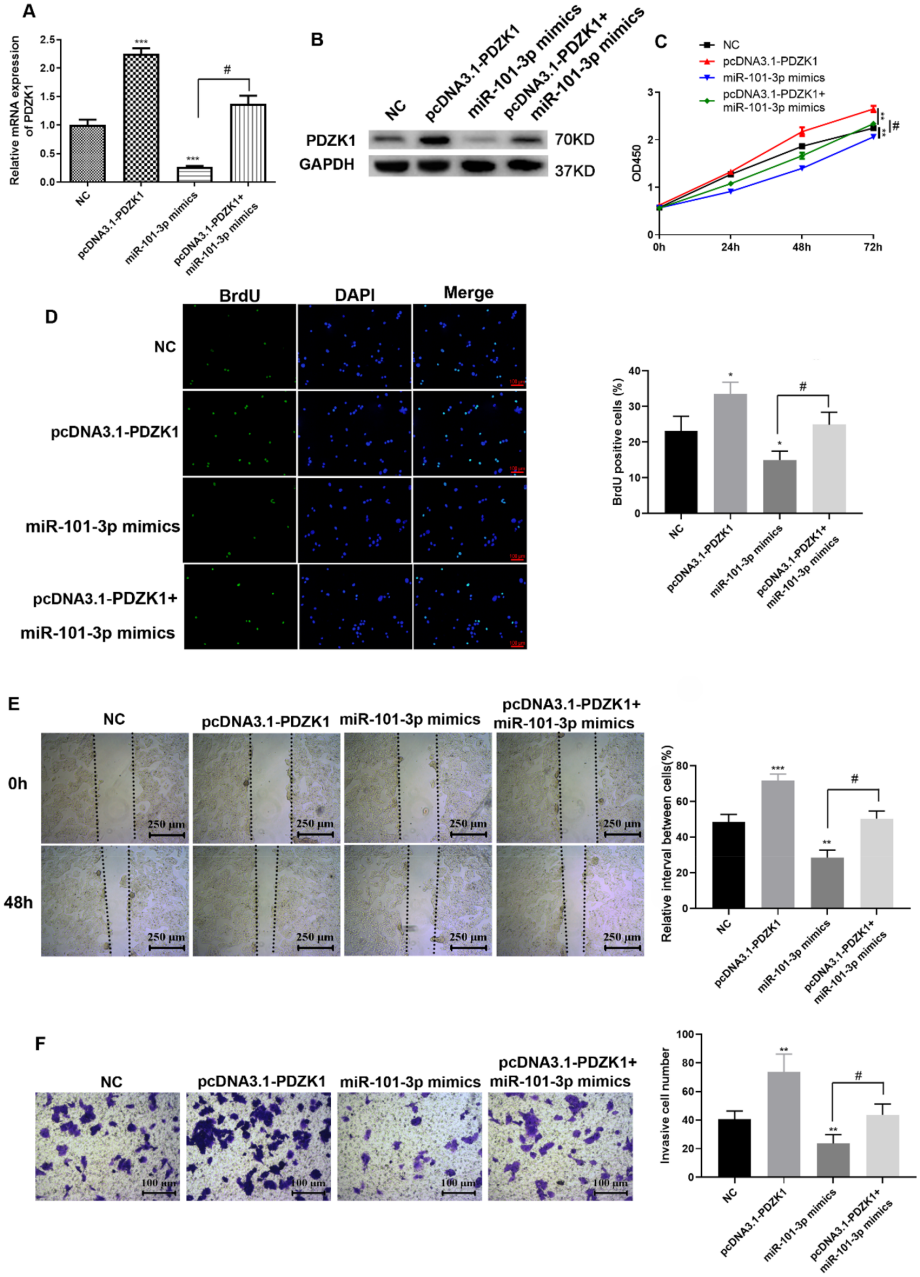
miR-101-3p-mediated proliferation, migration, and invasion of Huh7 cells via targeting of PDZK1. Transfection efficiency determined by qRT-PCR (**A**) and western blot analysis (**B**). MTT assay (**C**) and BrdU incorporation assays **D** were performed to determine cell proliferation. **E** Scratch assay of Huh-7 cell migration. **F** Transwell assay of Huh-7 cell invasion. Data are expressed as the mean ± SD values (n = 3). *P < 0.05, ***P < 0.001 compared to the NC group. #*P* < 0.05, ##*P* < 0.01 compared to the pcDNA 3.1-PDZK1 group

Rescue experiments were conducted to explore the interaction between PDZK1 and miR-101-3p in HCC. First, the transfection efficiency of miR-101-3p mimics was determined. As shown in Fig. 7A, B, miR-101-3p mimics could downregulate PDZK1 expression. In addition, transfection with miR-101-3p mimics was found to reduce the increase in PDZK1 expression, which was reversed by pcDNA3.1-PDZK1. According to the results of the MTT, BrdU, and Transwell assays, the miR-101-3p mimics significantly suppressed HCC cell proliferation and reduced the proliferation (Fig. 7C, D), migration (Fig. 7E), and invasion (Fig. 7F) abilities of Huh7 cells, which were reversed by pcDNA3.1-PDZK1 transfection. Thus, PDZK1 reversed the influence of miR-101-3p mimics on HCC cell proliferation, migration, and invasion. Taken together, these findings imply that miR-101-3p and PDZK1 have an interactive effect on HCC cell function.

### Role of PDZK1 in HCC mediated via the PI3K/AKT pathway

**Fig. 8:**
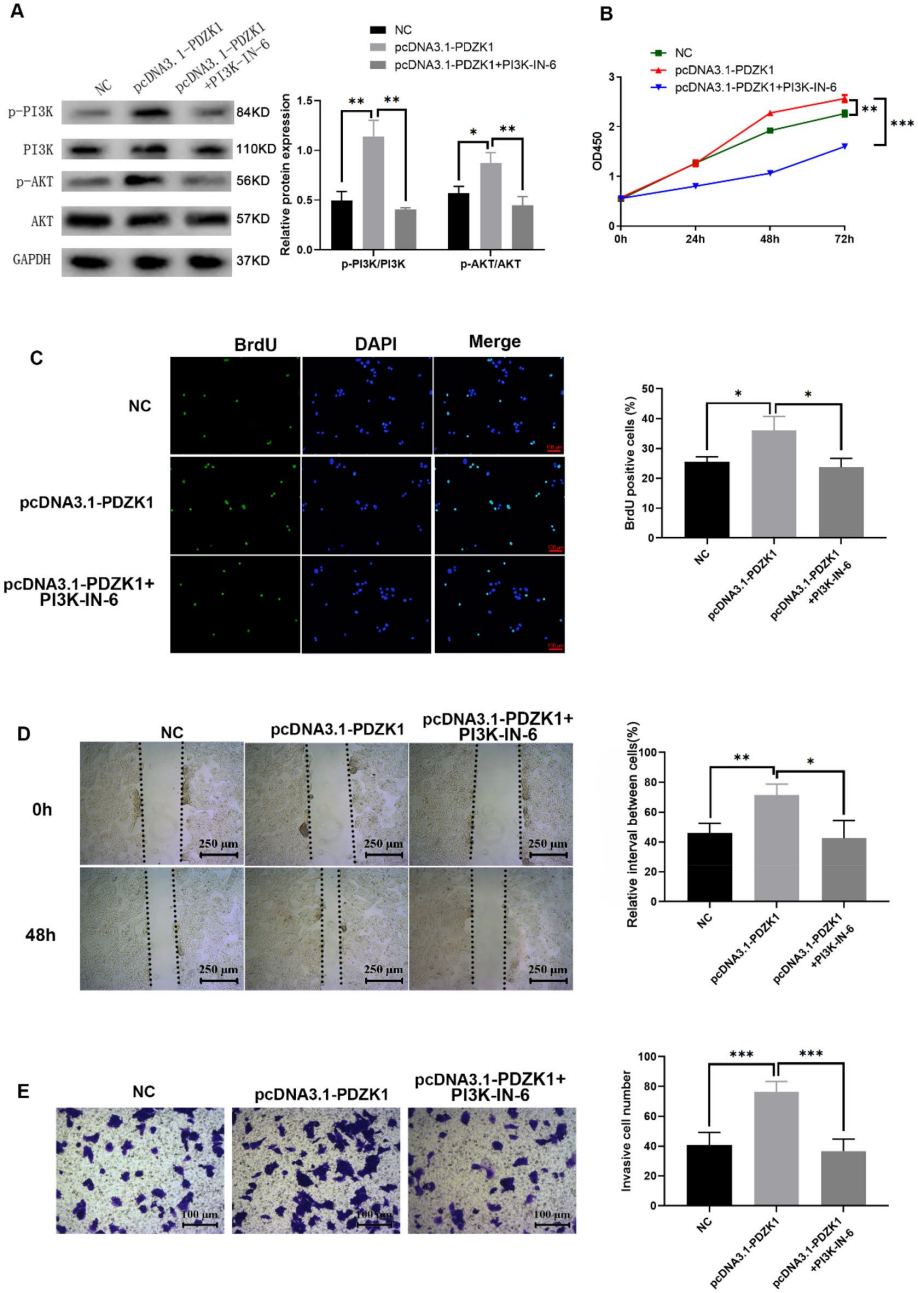
Role of PDZK1 in HCC mediated via the PI3K/AKT pathway. **A** Protein expression of p-PI3K, PI3K, p-AKT, and AKT under different experimental conditions. MTT assay (**B**) and BrdU incorporation assays (**C**) to determine cell proliferation. **D** Scratch assay of HCC cell migration. **E** Transwell assay of HCC cell invasion. Data are expressed as the mean ± SD values (n = 3). *P < 0.05, **P < 0.01, ***P < 0.001 compared to the NC group

Rescue experiments were conducted to confirm that PDZK1 played a role in HCC via the PI3K/AKT pathway. PI3K-IN-6, as a potent PI3K inhibitor, was used for inactivation of the PI3K/AKT pathway. As demonstrated by the western blot results in Fig. 8A, PI3K-IN-6 could significantly reduce the phosphorylation levels of PI3K and AKT that were elevated by PDZK1. The increased proliferative ability of HCC cells induced by pcDNA3.1-PDZK1 was also remarkably inhibited by PI3K-IN-6 according to the results of the MTT and BrdU assays (Fig. 8B, C). PI3K-IN-6 was found to have a similar effect on the migratory (Fig. 8D) and invasive (Fig. 8E) abilities of PDZK1-overexpression Huh7 cells. These findings suggest that PDZK1 plays a role in HCC via the PI3K/AKT pathway and inhibition of PDZK1 resulted in decreased cell proliferation and malignant progression of HCC.

## Discussion

Aberrant PDZK1 expression has been observed in several kinds of cancers. However, the role of PDZK1 in HCC is largely unknown. In the present study, we have explored the interaction between PDZK1 and miR-101-3p in HCC tissues and cells and the associated effects on HCC progression. The qRT-PCR results of the present study and HCC data deposited in the Starbase database demonstrated that PDZK1 expression was significantly higher in HCC tissues than in paracancerous and normal tissues. Further, the level of PDZK1 in HCC tissues was positively correlated with clinical stage, degree of differentiation of the tumor, and lymph node metastasis. In agreement with the findings of our study, Guo et al. used the publicly available online TGCA database and showed that the expression of PDZK1 is increased in HCC tissues compared with normal tissues and increases with cancer stage and tumor grade [30]. Further, the Kaplan–Meier analysis results demonstrated that the prognosis of HCC with high PDZK1 expression is poor [30]. These data suggest that PDZK1 might be a potential marker of HCC.

Based on the above studies, we have proved that PDZK1 was up-regulated in various HCC cells compared with LO2 cell, PDZK1-knockdown MHCC-97H cells and PDZK1-overexpression Huh7 cells were used for subsequent functional experiments and related pathway studies. The results of MTT and BrdU experiments in the present study demonstrated that PDZK1 can promote the proliferation of HCC cells, and scratch experiments and Transwell assays showed that PDZK1 accelerated the migration and invasion of HCC cells. These findings indicate that high PDZK1 expression in HCC cells promotes HCC progression. Previous studies have revealed that PDZK1 is an oncogene for tumorigenesis and the development of papillary thyroid cancer and esophageal cancer [20, 31]. On the other hand, Zheng et al. reported that low level of PDZK1 predicts poor clinical outcome in patients with clear cell renal cell carcinoma [32], and this suggests that PDZK1 may play different biological roles in different cancers. All these findings imply the potential of PDZK1 as a marker of HCC progression and malignancy.

The cell cycle is a regulatory network that controls the sequence and timing of cell cycle events. There are three major cell cycle phases: the G1/S, G2/M, and S phases [33]. Flow cytometry analysis in the present study showed that the percentage of cells in the G0/G1 phase was increased after PDZK1 knockdown, while the distribution of cells in the G2/M and S phases was decreased. In contrast, PDZK1 overexpression had the opposite effect. This suggests that low expression of PDZK1 inhibits the cell cycle progression of liver cancer cells and, therefore, tumor progression.

The PI3K/AKT pathway is in a state of activation and is closely associated with cancer progression and the regulation of cancer cell proliferation and tumorigenesis [34, 35]. Western blot analysis in the present study confirmed that the expression of PDZK1 was correlated with the phosphorylation of AKT and PI3K, and demonstrated that the high expression of PDZK1 activated the phosphorylation of AKT and PI3K. We also found that PDZK1 played a role in HCC via the PI3K/AKT pathway and resulted in decreased cell proliferation and malignant progression of HCC.

As miRNAs have emerged as key factors associated with the development of tumors, the Starbase, TargetScan, and miRDB databases were scanned for miRNA targets of PDZK1 and miR-101-3p was identified as a target. It has been reported that miR-101-3p targets CUL4B to block the PI3K/AKT/mTOR pathway in prostate cancer cells [36]. In the present study, low expression of miR-101-3p was detected in the HCC tissues and HCC cells. Further, miR-101-3p mimics were found to suppress HCC cell proliferation, migration, and invasion, which were reversed by PDZK1. These findings imply that miR-101-3p targets PDZK1 in HCC and, thereby, deepens our understanding of the role of miR-101-3p in cancers.

Taken together, the results of our study show that high expression of PDZK1 is associated with lymph node metastasis, degree of tumor differentiation, and clinical stage of HCC. The result further demonstrate that the miR-101-3p/PDZK1 axis plays a role in regulating the proliferation, migration, and invasion ability of HCC cells by modulation of the PIK/AKT pathway, and suggest that miR-101-3p/PDZK1 may be a new therapeutic target for HCC. However, further cellular and animal experiments are required to explore the correlation between the miR-101-3p/PDZK1 axis and the occurrence and development of HCC, as well as the precise underlying molecular mechanism. Such research in the future can help identify new targets and treatment strategies for HCC.

## Conclusion

The findings of our study indicate that the miR-101-3p/PDZK1 axis plays a role in HCC and may be a novel biomarker of HCC progression and a new therapeutic target for HCC treatment.

## Methods

### Patient samples

HCC tissues and paracancerous tissues (that is, tissue within the range of 0.5–1 cm around the tumor) were acquired from 30 HCC patients diagnosed at the Second Affiliated Hospital of Jiaxing University between January 2015 and January 2016. This project received the approval of the Ethics Committee of the Second Affiliated Hospital of Jiaxing University and was conducted in accordance with the tenets of the 1964 Helsinki Declaration. All the patients signed informed consent forms before participating in this study.

### RNA extraction and qRT⁃PCR

Quantitative real-time reverse transcription PCR was used to detect PDZK1 expression as described in Min Luo et al. [37]. The primers were obtained from Sangon Biotech Co. Ltd. (China), and the sequences were as follows: PDZK1, F: 5′‐GAATGGGGTGAATGTGCTAGATG‐3′, R: 5′‐CCAGGGAGGAAACAATAGGGA‐3′; GAPDH, F: 5′‐CTTTGGTATCGTGGAAGGACTC‐3′, R: 5′‐GTAGAGGCAGGGATGATGTTCT‐3′. miR-101-3p, F: 5′-TGCGGCTACAGTACTGTGATA-3′, R: 5′-GTGCAGGGTCCGAGGT-3′, U6, F: 5′-CTCGCTTCGGCAGCACA-3′, R: 5′-AACGCTTCACGAATTTGCGT-3′. Data analysis was performed using the 2^⁃ΔΔCt^ relative quantification method.

### Western blot analysis

Briefly, RIPA buffer (Beyotime Biotechnology, China) was used to extract proteins, which were then quantitated with the bicinchoninic assay kit (Beyotime Biotechnology, China). The proteins (20 µg) were separated using 10% sodium dodecyl sulfate–polyacrylamide gel electrophoresis (Genscript, China) and then transferred to a polyvinyl difluoride membrane (Millipore, USA). Later, the membrane was blocked with Tris-buffered saline (TBS) and 0.1% Tween 20 (TBS-T) containing 5% bovine serum albumin for 2 h at 37 °C. The membrane was incubated with rabbit polyclonal anti-PDZK1 (1:100, 10507-2-AP; PROTEINTECH, USA), anti-PI3K (1:2000, ab32089; Abcam, USA), anti-phospho-PI3K (Y-607) (1:1000, ab278545; Abcam), anti-AKT (1:10000, ab8805; Abcam), anti-phospho-AKT (1:800, ab38449; Abcam), and anti-GAPDH (1:10000, 60004-1-Ig; PROTEINTECH) antibodies at 4℃ overnight and then with horseradish peroxide-conjugated IgG (1:2000, SAB90100H, SAB90200H; FRDBIO, China) at 37 ℃ for 1 h. Next, a chemiluminescence assay was performed with the ECL kit (Millipore, USA). GAPDH was used as an internal control to normalize the expression of the assayed proteins. The results of immunoblot analysis were quantitated with Image J.

### Immunohistochemical staining

Slices of HCC and normal tissue were dewaxed, dehydrated, and rehydrated. Primary antibody against PDZK1(10507-2-AP, PROTEINTECH, USA) was added to the sections and incubated overnight at 4 °C. Then, biotinylated secondary antibody (F2761; Thermo Fisher Scientific, Waltham, MA, USA) was applied according to the protocol of the SP-immunohistochemistry (IHC) test [38].

### Cell lines and transfections

The human normal liver cell line LO2 and the HCC cell lines MHCC-97H, MHCC-97L, Huh7, and HCCLM3 were purchased from Shanghai Institute for Biological Sciences. These cell lines are free from mycoplasma contamination and were validated by short tandem repeat (STR) profiling within 5 years. Except for the MHCC-97L/H cell line, the STR profiles of the other cell lines were matched with the published reference map (DSMZ database). Since the MHCC-97L/H cell line has not been stored in the cell bank, the STR profiles of the MHCC-97L/H cell line do not match other STR data in the database. The cells were cultured in Dulbecco modified Eagle medium at 37 °C in an incubator with a 5% volume fraction of CO_2_ and saturated humidity.

siRNA against PDZK1 was designed based on previously published literature [21]: sense: 5′-CAAAGAAACUGACAAGCGUdTdT-3′, anti-sense: 5′-ACGCUUGUCAGUUUCUUUGdTdT-3′. si-PDZK1, full-length PDZK1, an miR-101-3p mimic, and the corresponding negative controls were obtained from Sangon Biotech Shanghai (China), and the related sequence primer were as shown in Additional file 1. When the Huh7 cells in each well reached a confluence rate of 60–80%, transfection procedures were performed with Lipofectamine 2000 (11668-019, Invitrogen) according to the manufacturer’s instructions. The PI3K-IN-6 (MCE, HY-101115; 5 nM) was added after pcDNA3.1-PDZK1 transfection at different time points to detect cell proliferation activity, and in 48 h detection ability of cell proliferation and migration.

### Cell proliferation assay

The MTT assay was utilized to evaluate cell proliferation, with 1 × 10^4^ cells seeded per well. The cells were treated with 5 μg/μL MTT solution at 0, 24, 48, and 72 h after transfection and incubated for 4 h. After incubation, the medium was discarded, and DMSO was added to terminate the reaction. Absorption was measured at a wavelength of 570 nm with a microplate reader.

Bromodeoxyuridine (BrdU) incorporation experiments were also used to determine cell proliferation. After 48 h of cell transfection, cells were incubated for 4 h, fixed with 4% paraformaldehyde, and stained with BrdU and DAPI antibodies provided with the BrdU kits (ST1056-100 mg; Beyotime, China). The experiments were conducted in triplicate, and the BrdU-positive rate was calculated as the number of BrdU-stained cells divided by the number of DAPI-stained cells.

### Scratch test

Transfected Huh7 and MHCC-97H cells were inoculated at a density of 1 × 104 cells per well. When the cells reached confluence (90–100%), scratches were made on the surface of the culture, and then the cells were cultured in FBS-free medium [39]. After 0 and 48 h, images of the scratched area were obtained from five fields of view. The scratched area was measured using the Image J software, and the relative interval between cells was calculated.

### Cell invasion assay

The cell invasion assay was performed in triplicate using 24-well Transwell chambers (no. 3422; Corning, USA) based on the manufacturer’s instructions and previously reported protocols [37]. After 24 h, the cells on the underside of the inserts were fixed in methanol for 10 min and stained with 0.1% crystal violet. Only the stained cells are the ones that go through the Matrigel.

### Apoptosis and cell cycle assay

Transfected cells were seeded at a density of 1 × 10^4^ cells per well. After the cells were attached, they were stained for apoptosis and cell cycle detection based on the instructions of the Annexin-V FITC/PI apoptosis detection kit (40302ES20, Yeasen; Shanghai, China) and cell cycle staining kit (40302ES50, Yeasen) separately. After staining, analysis was conducted using a FACSVerse flow cytometer (Becton Dickinson, CA).

### Luciferase reporter assay

Wild-type (WT) PDZK1 3′-UTR and mutant (MUT) PDZK1 3′-UTR oligonucleotides containing the putative binding site for miR-101-3p were cloned into the luciferase-expressing pGL3 vector (GenePharma Co., Ltd, China). The sequences of oligo PDZK1 3′-UTR and mutant (MUT) PDZK1 3′-UTR were shown in Additional file 2. Following this, WT-PDZK1 3′-UTR or Mut-PDZK1 3′-UTR were co-transfected with the miR-101-3p mimic or miRNA negative control (miR-NC) into 293 T cells (1 × 10^5^ cells/well) in 48-well plates, using the Lipofectamine® 2000 reagent. The relative luciferase activity was determined using a Dual-Luciferase Reporter assay system (RG043S, Beyotime) after transfection for 48 h according to the manufacturer’s protocol. The firefly luciferase activity was normalized to Renilla luciferase activity.The sequences of oligo PDZK1 3′-UTR and mutant (MUT) PDZK1 3′-UTR was shown in Additional file 2.

### Bioinformatics prediction

The online bioinformatics analysis websites Starbase (https://starbase.sysu.edu.cn) [40], TargetScan (http://www.targetscan.org) [41], and miRDB (http://mirdb.org) [42] were used to predict miRNA binding to miR-101-3p.

### Statistical analysis

Data were analyzed with Graphpad Prism 7.0 (Graphpad, USA) or SPSS11.0 (SPSS Inc., USA) and presented as the mean ± SD value from three independent experiments. The chi‐square test was used to evaluate the influence of PDZK1 on the clinicopathological characteristics of HCC. Survival curves were produced with the Kaplan–Meier method. Student’s *t*-test or one-way ANOVA was used for statistical comparison between groups. *P* < 0.05 was considered to indicate statistically significant differences.

### Supplementary Information

**Additional file 1: The expression levels of miR-30a-5p, miR-2114-3p and miR-101-3p: Figure S1.** The expression levels of miR-30a-5p, miR-2114-3p and miR-101-3p. The levels of miR-30a-5p in Starbase samples (**A**) and samples from our hospital (**B**); The levels of miR-2114-3p in Starbase samples (**C**) and samples from our hospital (**D**); The levels of miR-101-3p in Starbase samples (**E**) and samples from our hospital (**F**). **P < 0.01, ***P < 0.001, ns means no significance.

**Additional file 2: Additional information;** The si-PDZK1, full-length PDZK1, and an miR-101-3p mimic sequences; The sequences of oligo PDZK1 3’-UTR and mutant (MUT) PDZK1 3’-UTR.

## Data Availability

All the data generated in this study can be provided by the corresponding author upon reasonable request.
