# Supplementary material for: miR-101-3p-mediated role of PDZK1 in hepatocellular carcinoma progression and the underlying PI3K/Akt signaling mechanism
Source: Cell Div. 2024 Mar 26;19:9. doi: 10.1186/s13008-023-00106-6 (PMC10964575; doi:10.1186/s13008-023-00106-6)
Supplement: Supplementary file 1 — Additional file 1: The expression levels of miR-30a-5p, miR-2114-3p and miR-101-3p: Figure S1. The expression levels of miR-30a-5p, miR-2114-3p and miR-101-3p. The levels of miR-30a-5p in Starbase samples (A) and samples from our hospital (B); The levels of miR-2114-3p in Starbase samples (C) and samples from our hospital (D); The levels of miR-101-3p in Starbase samples (E) and samples from our hospital (F). **P < 0.01, ***P < 0.001, ns means no significance. [file 13008_2023_106_MOESM1_ESM.docx]

**
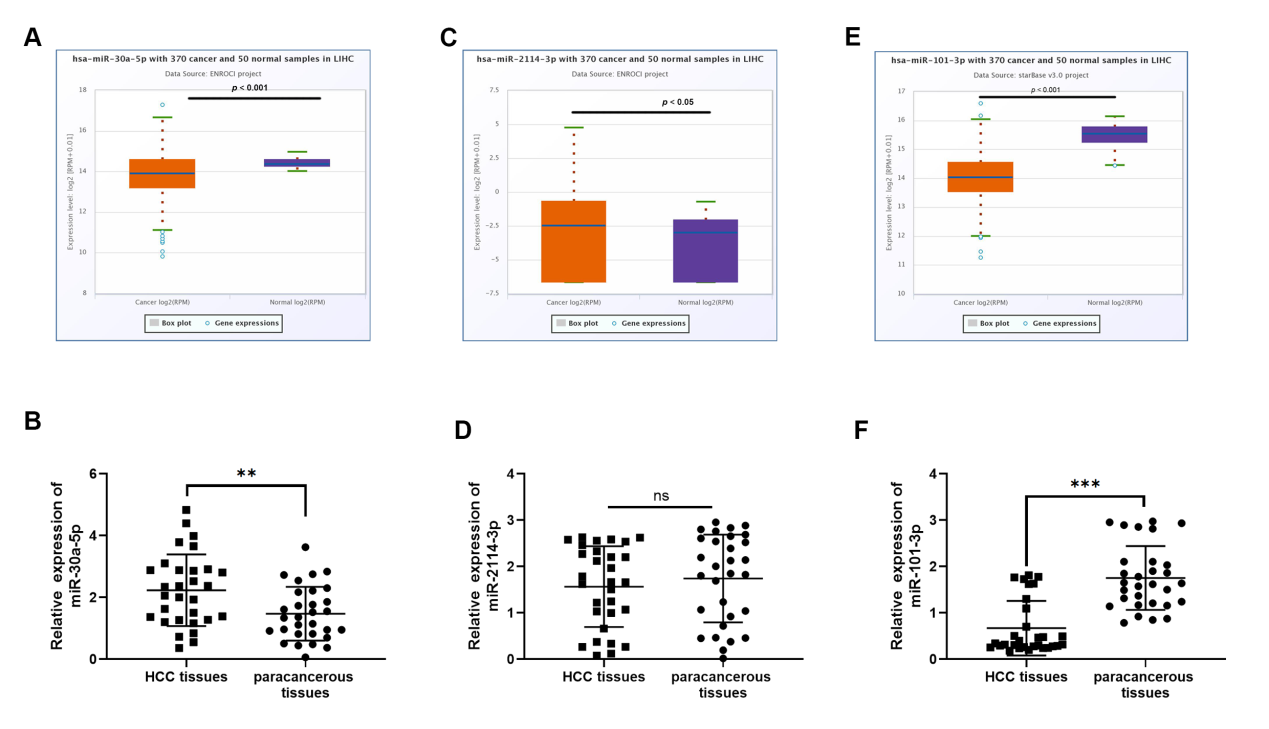
Additional File 1:** Figure S1. The expression levels of miR-30a-5p, miR-2114-3p and miR-101-3p. The levels of miR-30a-5p in Starbase samples (A) and samples from our hospital (B); The levels of miR-2114-3p in Starbase samples (C) and samples from our hospital (D); The levels of miR-101-3p in Starbase samples (E) and samples from our hospital (F). **P < 0.01, ***P < 0.001, ns means no significance.
