# Supplementary material for: miR-101-3p-mediated role of PDZK1 in hepatocellular carcinoma progression and the underlying PI3K/Akt signaling mechanism
Source: Cell Div. 2024 Mar 26;19:9. doi: 10.1186/s13008-023-00106-6 (PMC10964575; doi:10.1186/s13008-023-00106-6)
Supplement: Supplementary file 2 — Additional file 2: Additional information; The si-PDZK1, full-length PDZK1, and an miR-101-3p mimic sequences; The sequences of oligo PDZK1 3’-UTR and mutant (MUT) PDZK1 3’-UTR. [file 13008_2023_106_MOESM2_ESM.docx]

**Additional File 2** Additional information; The si-PDZK1, full-length PDZK1, and an miR-101-3p mimic sequences were as follows:

Si-PDZK1: Sense: GGUGAAUGUGCUAGAUGAATT; Antisense: UUCAUCUAGCACAUUCACCTT; **pcDNA3.1-PDZK1:**ATGACCTCCACCTTCAACCCCCGAGAATGTAAACTGTCCAAGCAAGAAGGGCAAAACTATGGCTTCTTCCTGCGAATTGAGAAGGACACCGAGGGCCACCTGGTCCGGGTGGTTGAGAAGTGTAGCCCAGCAGAGAAGGCTGGCCTTCAAGATGGAGACAGAGTTCTTAGGATCAATGGTGTCTTTGTGGACAAAGAAGAACATATGCAGGTTGTGGATCTGGTCAGAAAGAGTGGGAATTCAGTGACTTTACTAGTTCTGGATGGGGATTCCTATGAGAAAGCAGTGAAAACACGGGTGGACTTGAAAGAGTTGGGTCAAAGTCAGAAGGAGCAAGGTTTGAGTGATAATATACTTTCCCCTGTGATGAATGGAGGTGTGCAAACTTGGACCCAGCCCCGGCTCTGCTATCTCGTGAAGGAAGGAGGCAGCTATGGCTTCTCTCTGAAAACTGTCCAAGGTAAAAAGGGGGTGTACATGACTGATATTACACCTCAAGGTGTGGCTATGAGAGCTGGAGTTCTGGCTGATGATCACTTGATTGAAGTGAATGGAGAGAATGTAGAGGATGCCAGCCATGAGGAAGTGGTTGAAAAGGTGAAGAAGTCAGGAAGCCGTGTCATGTTCCTGCTGGTGGACAAAGAAACTGACAAGCGTCATGTTGAGCAGAAGATACAATTCAAAAGAGAAACAGCCAGTTTGAAACTGTTACCCCACCAGCCCCGAATTGTGGAGATGAAGAAAGGAAGCAATGGCTATGGTTTCTATCTGAGGGCAGGCTCAGAACAGAAAGGTCAAATCATCAAGGACATAGATTCTGGAAGTCCAGCAGAGGAGGCTGGCTTGAAGAACAATGATCTGGTAGTTGCTGTCAACGGCGAGTCTGTGGAAACCCTGGATCATGACAGTGTGGTAGAAATGATTAGAAAGGGTGGAGATCAGACTTCACTGTTGGTGGTAGACAAAGAGACGGACAACATGTACAGACTGGCTCATTTTTCTCCATTTCTCTACTATCAAAGTCAAGAACTGCCCAATGGCTCTGTCAAGGAGGCTCCAGCTCCTACTCCCACTTCTCTGGAAGTCTCAAGTCCACCAGATACTACAGAGGAAGTAGATCATAAGCCTAAACTCTGCAGGCTGGCTAAAGGTGAAAATGGCTATGGCTTTCACTTAAATGCGATTCGGGGTCTGCCAGGCTCATTCATCAAAGAGGTACAGAAGGGCGGTCCTGCTGACTTGGCTGGGCTAGAGGATGAGGATGTCATCATTGAAGTGAATGGGGTGAATGTGCTAGATGAACCCTATGAGAAGGTGGTGGATAGAATCCAGAGCAGTGGGAAGAATGTCACACTTCTAGTCTGTGGAAAGAAGGCCTATGATTATTTCCAAGCTAAGAAAATCCCTATTGTTTCCTCCCTGGCTGATCCACTTGACACCCCTCCAGATTCTAAAGAAGGAATAGTGGTGGAGTCAAACCATGACTCGCACATGGCAAAAGAACGGGCCCACAGTACAGCCTCACATTCTTCTTCCAATTCTGAAGATACAGAGATGTGA; MiR-101-3p mimic: 5’-UACAGUACUGUGAUAACUGAA-3’; 5’-CAGUUAUCACAGUACUGUAUU-3’; Negative control: 5’-UUCUCCCGAACGUGUCACGUTT-3’; 5’-ACGUGACACGUUCGGAGAATT-3’

The sequences of oligo PDZK1 3’-UTR and mutant (MUT) PDZK1 3’-UTR was as follows:

PDZK1 3’UTR，WT:

TGAAAACAAGTAATAGCTTTGGCTGTTTATTTGATAGCTGTTTCTGGGTATTTAATAGGAATCCTTTCTCAAGGAATGAGTTGTGACCTGTTTACTGTCTCTTTAGAAGAAAAACTCCACTGGAAACCATTCACCATGTGTGATTGTCTTCTGTTATCATTTGTCTTACAGGCGGCTATTGCAGACGGCTAATTTATGCTTAACTTAGGAAGAGATAAGGCAAGAGCTAGATTTTTTTCATGTGATCTTTTCCAAGCTTCAACTTAACTTAACTACATTTCTCTGTATGATGATGTCTCTTACTTCTACAGGTTCCTTGAGCACCAAAGATGATTCATAACTCTGTATAGGTGACAGCTGCTTATAAAAGCATCTTAGCAGATAAGCCTATTAAAATTGTGCTTTTGTAACAATGTTGTGGTTGCTAGAATAAATACCATTAACAAATGCCTTTTGAGTATGCTTGATAGTGCTTTTGTTTTGGATTCACTTTTTATGCTTTAACCTTCATTTGCCTCTAGAAACCCAAAACACAATAAAGTACAGAATAAGACCTTAGTAATAAAATTCAGAATTTTCTTAAA; PDZK1 3’UTR mut:

TGAAAACAAGTAATAGCTTTGGCTGTTTATTTGATAGCTGTTTCTGGGTATTTAATAGGAATCCTTTCTCAAGGAATGGACCGTGACCTGTTCGTCACCTCTTTAGAAGAAAAACTCCACTGGAAACCATTCACCATGTGTGATTGTCTTCTGTTATCATTTGTCTTACAGGCGGCTATTGCAGACGGCTAATTTATGCTTAACTTAGGAAGAGATAAGGCAAGAGCTAGATTTTTTTCATGTGATCTTTTCCAAGCTTCAACTTAACTTAACTACATTTCTCTGTATGATGATGTCTCTTACTTCTACAGGTTCCTTGAGCACCAAAGATGATTCATAACTCTGTATAGGTGACAGCTGCTTATAAAAGCATCTTAGCAGATAAGCCTATTAAAATTGTGCTTTTGTAACAATGTTGTGGTTGCTAGAATAAATACCATTAACAAATGCCTTTTGAGTATGCTTGATAGTGCTTTTGTTTTGGATTCACTTTTTATGCTTTAACCTTCATTTGCCTCTAGAAACCCAAAACACAATAAAGTACAGAATAAGACCTTAGTAATAAAATTCAGAATTTTCTTAAA
